# Imaging Pitfalls of Multilocular Cystic Renal Neoplasm of Low Malignant Potential: A Case Report

**DOI:** 10.7759/cureus.87249

**Published:** 2025-07-03

**Authors:** Yusuke Kaneko, Mako Ueno, Seiya Hattori, Mami Hatano, Shigeo Okuda

**Affiliations:** 1 Diagnostic Radiology, National Hospital Organization Tokyo Medical Center, Tokyo, JPN; 2 Urology, National Hospital Organization Tokyo Medical Center, Tokyo, JPN; 3 Pathology, National Hospital Organization Tokyo Medical Center, Tokyo, JPN

**Keywords:** imaging pitfall, mcrnlmp, renal cystic tumor, renal mass imaging, renal neoplasm

## Abstract

Multilocular cystic renal neoplasm of low malignant potential (MCRNLMP) is a rare subtype of renal tumor classified within the spectrum of clear cell renal tumors, and it is characterized by an excellent prognosis. Histologically, it is composed of multiple cysts lined by low-grade clear cells and separated by fibrous septa, with no solid expansile growth or significant nuclear atypia. Few reports have documented MCRNLMP mimicking a solid mass, especially in elderly patients. We report the case of an 80-year-old male in whom a left renal mass was incidentally detected during abdominal imaging performed as part of diabetes screening, and was ultimately diagnosed as MCRNLMP. Contrast-enhanced CT and MRI revealed a lesion in the mid-portion of the left kidney that appeared predominantly solid, raising strong suspicion for a malignant renal tumor. Robot-assisted nephrectomy (RAN) was performed, and postoperative histopathological analysis confirmed the diagnosis of MCRNLMP. The lesion showed prominent fibrous septa and intracystic hemorrhage, which likely contributed to its solid appearance on imaging. This report illustrates a diagnostic pitfall, where fibrous tissue may mimic solid tumor components on imaging, making it difficult to distinguish MCRNLMP from other solid renal tumors.

## Introduction

Multilocular cystic renal neoplasm of low malignant potential (MCRNLMP) is a rare subtype of renal tumor classified within the spectrum of clear cell renal tumors in the 2022 WHO classification. It accounts for approximately 1-2% of renal cell carcinomas [1]. Histologically, it is defined by the presence of multiple cysts lined by low-grade clear cells and separated by fibrous septa, with no expansile growth, solid nodules, or high-grade nuclear atypia [2,3]. MCRNLMP typically affects middle-aged to elderly individuals and is often discovered incidentally during imaging studies conducted for unrelated conditions [3].

Despite its excellent prognosis, preoperative diagnosis remains challenging as its imaging characteristics often resemble those of malignant cystic renal tumors, particularly cystic clear cell renal cell carcinoma (ccRCC) [4]. On cross-sectional imaging, MCRNLMP may be categorized as Bosniak IIF to IV, especially when septal or wall enhancement or solid-appearing components are present [5]. This overlap in radiologic presentation may lead to diagnostic uncertainty and, in some cases, overtreatment. Accurate identification of MCRNLMP is crucial to avoid unnecessary radical treatment. Radiologic-pathologic correlation and multidisciplinary collaboration are essential to differentiate MCRNLMP from more aggressive neoplasms. We present a case of MCRNLMP radiologically mimicking a solid malignant tumor, discuss the diagnostic pitfalls, and highlight the importance of careful interpretation of imaging findings.

## Case presentation

An 80-year-old male with a history of diabetes, hypertension, and old myocardial infarction was referred to our hospital following the incidental detection of a left renal mass during abdominal imaging conducted for screening purposes. The patient was asymptomatic, with no lower urinary tract symptoms or flank pain. The laboratory findings were unremarkable except for a mildly elevated HbA1c level of 7.6%; the serum creatinine was 0.79 mg/dL, and the estimated glomerular filtration rate (eGFR) was 71.4 mL/min/1.73 m². Urinalysis was unremarkable except for positive glycosuria.

**Figure 1:**
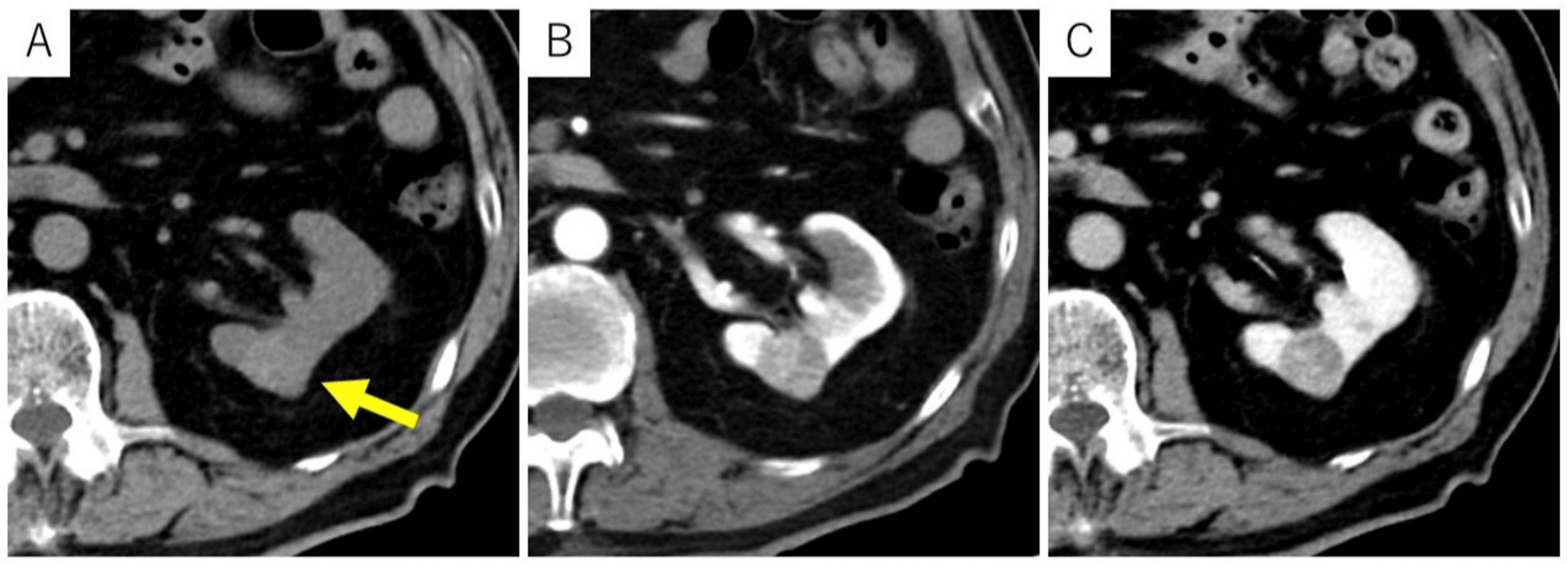
Dynamic CT of the abdomen On non-contrast CT (A), a round mass with slightly higher attenuation than the renal parenchyma was observed in the mid-portion of the left kidney. The lesion appeared predominantly solid, with internal low-attenuation areas suggesting cystic or degenerative components. The solid portion demonstrated mild enhancement in the early phase (B), followed by slight washout in the delayed phase (C) CT: computed tomography

**Figure 2:**
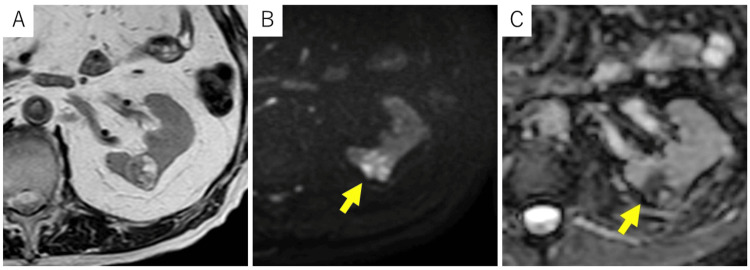
MRI of the abdomen A round mass was identified in the mid-portion of the left kidney. On T2-weighted imaging (A), the lesion showed a mixture of intermediate and high signal intensity areas. It was clearly demarcated from the surrounding normal renal parenchyma by a low-signal rim suggestive of a pseudocapsule. DWI (B) demonstrated diffusely high signal intensity throughout the lesion, while the ADC map (C) demonstrated a heterogeneous pattern with both low and high signal areas ADC: apparent diffusion coefficient; DWI: diffusion-weighted imaging; MRI: magnetic resonance imaging

CT revealed a well-defined 21-mm mass in the mid-portion of the left kidney (Figure 1). Although it contained a few small cystic structures, the lesion appeared predominantly solid, showing slightly higher attenuation than the renal parenchyma on non-contrast CT. It demonstrated mild enhancement, particularly on the right side of the tumor, measuring 134 Hounsfield units (HU) in the early phase and slight washout (103 HU) in the delayed phase. MRI characterized the lesion as a well-encapsulated solid mass (Figure 2). The solid component exhibited high signal intensity on diffusion-weighted imaging (DWI) and low values on the apparent diffusion coefficient (ADC) map, findings consistent with diffusion restriction. Additionally, areas of high signal intensity were partially observed on both T2-weighted imaging and DWI/ADC maps, suggesting the presence of degenerative changes or small cystic components. Based on these imaging findings, renal cell carcinoma was strongly suspected. Radical (total) nephrectomy was performed despite cT1a status; partial nephrectomy would be generally preferred, due to the patient’s advanced age, comorbidities including diabetes and prior myocardial infarction, and the increased risk of bleeding associated with anticoagulant therapy.

**Figure 3:**
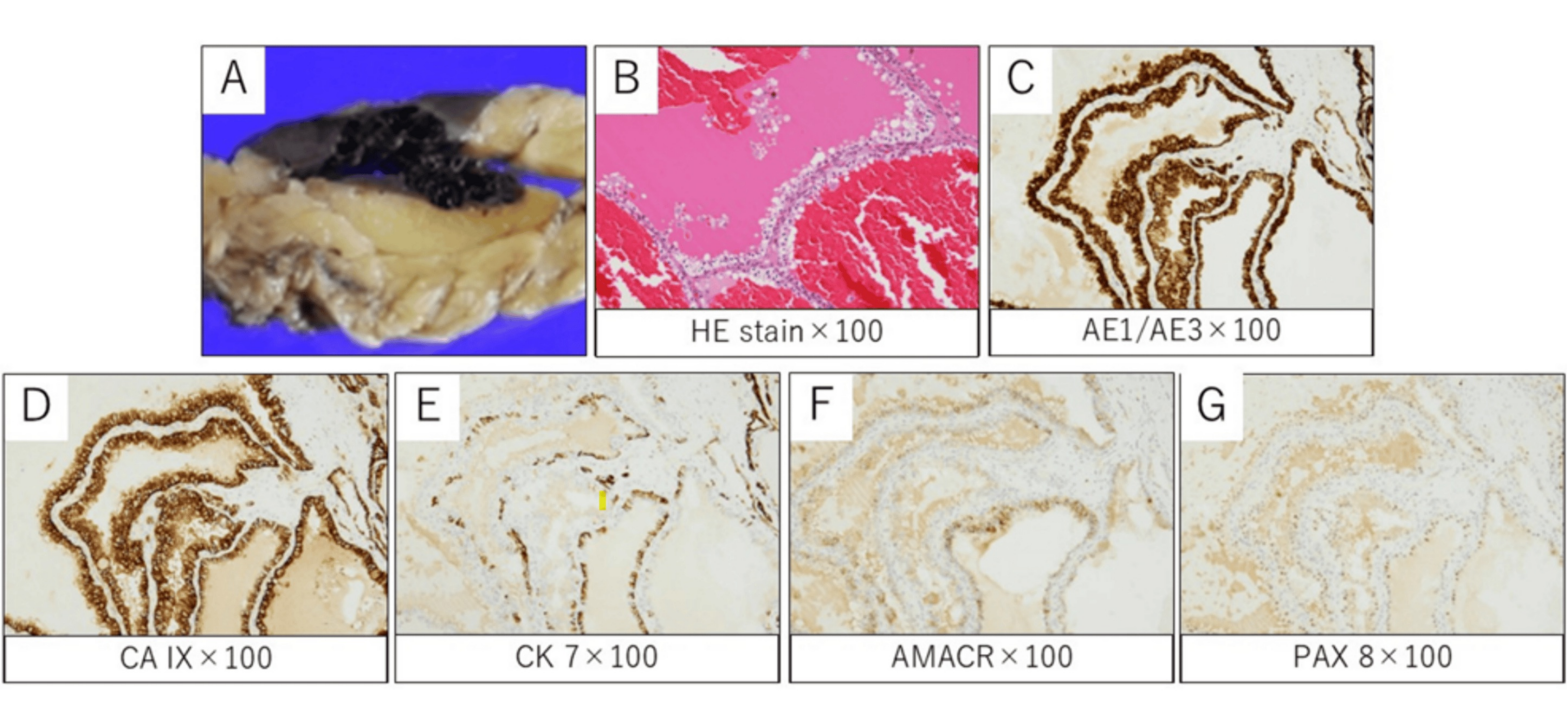
Pathological findings (A) Gross appearance of the resected specimen shows a well-circumscribed, brownish multilocular cystic tumor with focal hemorrhage. (B) Hematoxylin and eosin staining reveals cysts lined by clear cells without solid proliferation or high-grade atypia. Hemorrhage was diffusely observed throughout the tumor. Immunohistochemically, tumor cells were positive for (C) AE1/AE3, (D) CA IX, and focally positive for (E) CK7 and (F) AMACR. (G) PAX8 was weakly and focally positive. This immunoprofile supports that the present case falls within the spectrum of clear cell renal neoplasms. Collectively, the histological and immunohistochemical findings indicate a diagnosis of MCRNLMP MCRNLMP: multilocular cystic renal neoplasm of low malignant potential

Gross examination revealed a well-circumscribed, multilocular cystic lesion. Histologically, the tumor consisted entirely of multiple cysts lined by low-grade clear cells and separated by fibrous tissue. Notably, extensive hemorrhage was present within the cysts (Figure 3). The areas that appeared solid on imaging were correlated with fibrous septa and hemorrhagic cyst contents on histology, with no evidence of true neoplastic solid components. No expansile solid nodules or high-grade nuclear atypia were identified, confirming the final diagnosis of MCRNLMP. The postoperative course was uneventful, and the patient has remained recurrence-free for nine months following surgery.

## Discussion

MCRNLMP is a rare renal tumor characterized by an indolent clinical course and an excellent prognosis [6,7]. It typically appears as a multilocular cystic mass with septations and occasionally solid-appearing components. These solid areas are often composed of fibrous tissue and usually lack early contrast enhancement. However, due to overlapping imaging features with other cystic renal neoplasms, preoperative differentiation can be difficult, often leading to overestimation of malignancy and resulting in unnecessary surgical resection [7]. Recent studies have shown that up to 20% of surgically resected Bosniak III and IV lesions are diagnosed as benign on histopathological examination, underscoring the importance of cautious interpretation to avoid overtreatment [8]. Histologically, MCRNLMP is defined by the presence of multiple cysts lined by low-grade clear cells separated by fibrous septa, without expansile solid proliferation or high-grade nuclear atypia [2,3].

Typically, the differential diagnosis of cystic renal lesions includes cystic clear cell renal cell carcinoma (cystic ccRCC), tubulocystic renal cell carcinoma (tcRCC), and mixed epithelial and stromal tumor (MEST) [9]. Cystic ccRCC typically presents with thickened, irregular septa or mural nodules, showing early enhancement and washout on dynamic contrast imaging. These enhancing components usually reflect viable tumor tissue, in contrast to MCRNLMP, in which solid-appearing areas are often composed of fibrous tissue and generally lack early contrast enhancement. This difference in enhancement pattern may aid in distinguishing MCRNLMP from cystic ccRCC, although preoperative differentiation remains challenging.

TcRCC typically presents as a cystic renal mass categorized as Bosniak II to IV. It may resemble a simple renal cyst on CT, showing a well-defined, low-attenuation lesion with minimal enhancement. In contrast, ultrasound often reveals high echogenicity with posterior acoustic enhancement, creating a discrepancy between modalities. This discordance itself can be a diagnostic clue. MRI, particularly T2-weighted and SSFP sequences, offers superior tissue resolution and can clearly depict the lesion’s characteristic fine tubular and cystic structures, further supporting the diagnosis [10]. MEST typically occurs in middle-aged women and can occasionally protrude into the renal pelvis, presenting as a cystic tumor with solid components. In contrast, MCRNLMP has no specific predilection for location but predominantly occurs in middle-aged to older adults with a slight male predominance, differing in typical age and sex distribution [11].

In our patient, the lesion appeared predominantly solid on both CT and MRI. CT showed mild enhancement with soft tissue density, while MRI demonstrated areas of diffusion restriction. The lesion exhibited early enhancement and delayed washout, and based on this enhancement pattern, ccRCC and chromophobe RCC were initially considered in the differential diagnosis. However, retrospective pathological correlation revealed that these features reflected a combination of fibrous septa and intracystic hemorrhage rather than actual tumor proliferation. While thickening of fibrous septa is relatively common, the extensive hemorrhage involving the entire lesion, as observed in this case, is atypical. Renal mass biopsy is not routinely performed in all cases of suspected RCC. However, it may be considered in specific clinical scenarios where the diagnostic outcome could influence management. These include small renal masses in patients who are poor surgical candidates, unresectable or metastatic lesions, and tumors suspected to be of non-RCC origin. In such cases, a multidisciplinary approach involving radiologists, urologists, and pathologists is crucial to determine the appropriateness and safety of the procedure [12].

MCRNLMP is a renal tumor with an excellent prognosis, with no reported cases of recurrence or metastasis following complete resection. Accordingly, there is no clear consensus regarding the need for strict postoperative surveillance. A study by Li et al. suggested that extending the follow-up interval after surgery may be feasible, potentially minimizing unnecessary examinations and reducing patient burden [2].

This report highlights an important diagnostic pitfall: fibrous tissue and hemorrhage within cysts can mimic solid tumor components on imaging. Thus, awareness of this potential diagnostic pitfall is essential to avoid misinterpretation and unnecessary radical surgery. Upon retrospective review, the solid-appearing areas showed mildly increased attenuation on non-contrast CT, possibly reflecting intracystic hemorrhage. Careful evaluation of attenuation values on non-contrast CT might help avoid this diagnostic pitfall.

## Conclusions

While MCRNLMP is a rare renal tumor with an excellent prognosis, fibrous tissue or intracystic hemorrhage can mimic solid components on imaging, making differentiation from malignancy difficult. Slightly high attenuation on non-contrast CT may suggest hemorrhagic contents rather than a true tumor. Recognizing this pitfall is important to avoid overdiagnosis and unnecessary radical surgery in fragile patients.
